# The microbial contribution to macroecology

**DOI:** 10.3389/fmicb.2014.00203

**Published:** 2014-05-05

**Authors:** Albert Barberán, Emilio O. Casamayor, Noah Fierer

**Affiliations:** ^1^Cooperative Institute for Research in Environmental Sciences, University of ColoradoBoulder, CO, USA; ^2^Biogeodynamics and Biodiversity Group, Department of Continental Ecology, Center for Advanced Studies of Blanes – Spanish Council for Research (CSIC)Blanes, Spain; ^3^Department of Ecology and Evolutionary Biology, University of ColoradoBoulder, CO, USA

**Keywords:** macroecology, microbial ecology, dispersal, speciation, stochastic geometry, neutral theory

## Abstract

There has been a recent explosion of research within the field of microbial ecology that has been fueled, in part, by methodological improvements that make it feasible to characterize microbial communities to an extent that was inconceivable only a few years ago. Furthermore, there is increasing recognition within the field of ecology that microorganisms play a critical role in the health of organisms and ecosystems. Despite these developments, an important gap still persists between the theoretical framework of macroecology and microbial ecology. We highlight two idiosyncrasies of microorganisms that are fundamental to understanding macroecological patterns and their mechanistic drivers. First, high dispersal rates provide novel opportunities to test the relative importance of niche, stochastic, and historical processes in structuring biological communities. Second, high speciation rates potentially lead to the convergence of ecological and evolutionary time scales. After reviewing these unique aspects, we discuss strategies for improving the conceptual integration of microbes into macroecology. As examples, we discuss the use of phylogenetic ecology as an integrative approach to explore patterns across the tree of life. Then we demonstrate how two general theories of biodiversity (i.e., the recently developed theory of stochastic geometry and the neutral theory) can be adapted to microorganisms. We demonstrate how conceptual models that integrate evolutionary and ecological mechanisms can contribute to the unification of microbial ecology and macroecology.

## INTRODUCTION

Many of the important concepts driving both the theory and practice of ecological research were developed without explicit consideration of microorganisms, which represent the bulk of the phylogenetic and functional diversity on Earth. This omission is likely a consequence of the methodological difficulties associated with observing microbes in nature, and a product of the very different historical paths followed by the disciplines of microbiology and general ecology (Jessup et al., 2004; Prosser et al., 2007). While plant and animal ecologists have traditionally been influenced by more theoretical and holistic perspectives (Margalef, 1963), environmental microbiology, and microbial ecology have often relied on more reductionist experimental approaches (O’Malley and Dupré, 2007; Prosser et al., 2007). As suggested by O’Malley and Dupré (2007), an excessive focus on “macro”-organisms (i.e., plants and animals) may have distorted several basic aspects of our view of organismal ecology. With an ever-growing body of research focused on microbial ecology and biogeography (Martiny et al., 2006; Fierer, 2008; Hanson et al., 2012; Soininen, 2012), it is important to understand if the underlying ecological dynamics of plant and animal communities are fundamentally distinct from those observed in microbial communities. With the advent of DNA- and RNA-based techniques, microbial ecologists have been able to describe microbial diversity to an extent that was unimaginable only a few years ago (Curtis et al., 2006), and are now able to investigate the distribution of microorganisms in the environment and acquire detailed information on the phylogenetic and functional characteristics of microbial communities (Handelsman, 2004). Unfortunately, the rate of information collection by molecular techniques is far outpacing the rate at which researchers can properly analyze and interpret the data in an ecological context. Hence, in order to increase the understanding of highly diverse microbial communities embedded in a complex environmental milieu with ecological and evolutionary processes operating at multiple spatial and temporal scales, microbiologists can make use and expand concepts that have been developed in macroecology.

What is macroecology? The discipline of macroecology seeks to broaden the scope of ecology to much larger spatial and temporal scales by means of a comparative statistical methodology (Brown, 1995; Maurer, 1999). Thus, it attains greater potential for generalization and synthesis but with a less detailed delineation of the phenomenon under study (Brown, 1995). Typically, macroecologists explore patterns in the abundance of different species in a community (species abundance distributions); how the number of species (richness) varies with latitude, elevation and/or area, and the change in community similarity with spatial distance and/or environmental conditions (Brown, 1995; Maurer, 1999; Fierer, 2008; Soininen, 2012). Overall, macroecology acknowledges that no single mechanism explains trends across all scales, and that the scale of observation influences the patterns observed (Levin, 1992). Thus, one way to confront complexity is to adopt a more holistic point of view to circumvent the contingency of the specific organisms, communities, or ecosystems in question (Margalef, 1963; Maurer, 1999; Solé and Bascompte, 2006).

In their book The Microbe’s Contribution to Biology, based on a series of lectures given at Harvard University in 1954, Kluyver and van Niel (1956) demonstrated how microbiology could contribute to general biology. Unfortunately, the contributions of microbial ecologists to macroecology have been limited over the past 50 years, even though microbial communities could be considered to be ideally suited to research in macroecology as microbial data is essentially collected at a macroecological scale. First, microbial communities could expand the number of species and individuals included in macroecological datasets of plant and animal communities by several orders of magnitude (Whitman et al., 1998; Curtis et al., 2006). Second, large and relatively standardized datasets describing the phylogenetic and functional composition of microbial communities from a wide range of habitats have become publicly available to be explored and analyzed (Lozupone and Knight, 2007; Auguet et al., 2010). Finally, and perhaps most importantly, microbial systems allow experimental tests of macroecological hypotheses that would be very difficult to test with larger organisms (Jessup et al., 2004).

Here, we review two biological idiosyncrasies of microorganisms that are fundamental to understanding macroecological patterns and their mechanistic underpinnings in natural environments (we explicitly do not include any discussion on pathogenic microorganisms as they are more relevant to population ecology than to community ecology). First, as many microbes are likely capable of rapid, long-distance dispersal (Bovallius et al., 1978; Müller et al., 2013), this capacity for dispersal will likely influence the relative importance of niche, stochastic, and historical processes in shaping the structure of microbial communities. Second, rapid microbial evolution potentially leads to the convergence of ecological and evolutionary scales. After reviewing both microbial idiosyncrasies (acknowledging that these characteristics are also shared with some larger organisms and that not all microbes possess these shared characteristics) we show how microbes can be used to advance general concepts in macroecology. As examples of how this can be done, we discuss phylogenetic ecology as an integrative tool to explore patterns across the tree of life, and we demonstrate how the minimally sufficient rules of stochastic geometry (McGill, 2010) and the conceptual formulation of neutral theory (Hubbell, 2001) can be adapted to microorganisms.

## MICROBIAL IDIOSYNCRASIES AND MACROECOLOGY

### HIGH DISPERSIBILITY AND THE RELATIVE IMPORTANCE OF NICHE, STOCHASTIC, AND HISTORICAL PROCESSES IN STRUCTURING BIOLOGICAL COMMUNITIES

**FIGURE 1:**
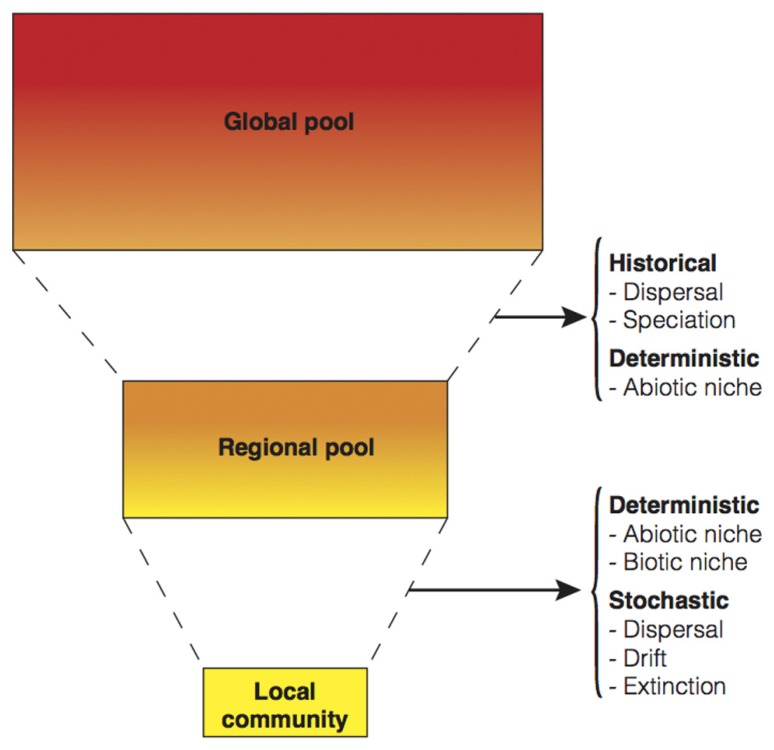
**Conceptual summary of the main processes influencing community composition, structure, and diversity at different spatial scales**. All ecological and evolutionary processes considered have been encapsulated in three perspectives: deterministic (i.e., the biotic and abiotic niche), stochastic, and historic. It can be argued that each one of the processes may have deterministic, stochastic, and historic components. For example, dispersal may be stochastic when rates depend solely on population size, deterministic when traits that affect arrival and establishment are considered, and also historic if the information of past events is available. Additionally, although the same process can operate at different scales, in this simplistic model as spatial scale increases historical processes tend to be more relevant, while at small local scale stochasticity can play an important role. As follows, speciation often requires geographic barriers, diverse niches and/or large population sizes to take place. On the contrary, the stochastic change in the abundance of organisms (drift) that can eventually result in extinction is more important at small population sizes. The demarcation of discrete spatial scales is arbitrary and will be dependent on the study system in question.

There are three basic perspectives on the dominant factors that influence the patterns of community diversity and composition (**Figure 1**). First, the classical deterministic niche-based perspective is based on the assumption that phenotypic attributes of species influence their interactions with other species and with the environment in predictable ways (Hutchinson, 1957). In contrast, the second perspective postulates that community assembly is largely based on stochastic processes. The recognition that chance can structure communities dates back to Grinnell (1922). He argued that finding rare species represented by a single individual can often be a result of fortuitous dispersal. The idea of stochasticity played a central role in the theory of island biogeography (MacArthur and Wilson, 1967), and gained new prominence with the unified neutral theory of biodiversity (Hubbell, 2001). Finally, the third perspective emphasizes the role of historical factors (notably, past speciation and former dispersal at the regional scale) over local processes in the assembly of communities (Ricklefs, 1987).

Microbial ecologists have relied almost exclusively on environmental explanations (i.e., niche-based perspective) to explain microbial community dynamics across time and space under the implicit assumption that everything is everywhere: but the environment selects (Baas Becking, 1934). This tenet does not mean that there are no biogeographical patterns, but rather it highlights that, due to high rates of microbial dispersal and large population sizes, geographic distance between habitats is usually thought to be irrelevant to community assembly. Disentangling the relative influence of niche, stochastic, and historical processes is one of the main tasks of community ecologists (**Figure 1**) and invariably all of these processes will influence communities with the relative importance of these processes varying depending on the spatial and temporal scale in question. However, local factors are in general more straightforward to measure, and historical events, such as past dispersal barriers or past environmental conditions, can only be detected in the context of spatial effects as usually there are no temporal records (Lindström and Langenheder, 2012). Communities of highly dispersive organisms like microbes have been shown to be less likely to exhibit signatures of spatial proximity and more likely to manifest the effects of the local environment in their community similarity patterns (Beisner et al., 2006; Lozupone and Knight, 2007; Auguet et al., 2010). However, there is also some evidence to suggest that microorganisms unique to rare or extreme habitats (like those found in hotsprings) can experience important dispersal barriers (Whitaker, 2006). Overall, we might expect microbes to show a wide range of patterns from true cosmopolitanism to endemism depending on the habitat and the spatial or taxonomic scales explored.

Although microbes likely have a relatively high capacity for dispersal (Bovallius et al., 1978; Müller et al., 2013), successful colonization requires both arrival and establishment. Asexual microorganisms tend to be excellent colonizers because of their dispersal capabilities and because even a single individual can potentially form a new local population (Brown, 1995). Dormancy (i.e., a reversible state of low metabolic activity) is also common in many microbial habitats, including soil, where it has been estimated that 80% of all microbial cells may be dormant at a given point in time (Lennon and Jones, 2011). Dormancy not only reduces some of the physiological limitations to dispersal, it also allows microbial taxa to persist when exposed to temporal variability in environmental conditions. Even a low dispersal rate combined with the capacity to remain viable during adverse conditions (via sporulation) might be enough to overcome dispersal limitation (De Meester, 2011). Thus, both dispersal and dormancy should reduce the risk of local extinction, and should increase the probability of successful colonization by avoiding mortality (Lennon and Jones, 2011). This concept is analogous to the seed bank in plant communities – a reservoir of genetic diversity that is capable of responding to environmental change, contributing to the diversity and dynamics of future generations (Lennon and Jones, 2011). The potentially high rates of dispersal and dormancy may partially account for the observation that many microbial communities have rank abundance curves with extremely long tails. However, there is some ongoing debate on the extent to which the large numbers of rare taxa reported from many communities may be a product of sequencing errors and/or heuristic processing algorithms (Kunin et al., 2010).

#### High speciation and the interplay between evolutionary and ecological scales

Microbial evolution can occur far more rapidly than the evolution of plants and animals, potentially leading to convergence of ecological and evolutionary time scales (Sniegowski et al., 1997; Denef and Banfield, 2012). It has been proposed that the large number of microbial species found in most environments is due to low extinction and high speciation rates (Dykhuizen, 1998). Although it is uncertain how evolution works in complex communities compared to laboratory cultures, it has been shown that rapid adaptation can actually occur in natural communities over a few decades (Denef and Banfield, 2012). However many barriers exists to minimize the horizontal exchange of genetic material (Thomas and Nielsen, 2005), it could be argued that horizontal gene transfer in diverse natural assemblages may act both as a diversifying (increasing the functional plasticity of the overall community) but also as a homogenizing force (leading to functional convergence among different species; Rosselló-Mora and Amann, 2001). The genetically isolated lineage, often conceived as the fundamental unit of evolution, may have no real analog in the asexual world (Rosselló-Mora and Amann, 2001), and hence most of life and its history cannot be simply conceived as an intelligible tree-like pattern (Maynard Smith et al., 1993). For this reason, definitions of what constitutes a bacterial species based on percentage DNA sequence similarity (a commonly used approach) could be considered somewhat arbitrary (Rosselló-Mora and Amann, 2001). As an alternative to the biological species concept for asexual microorganisms, the ecological species concept defines a species as a set of individuals showing genetic cohesion with shared ecological properties (Cohan and Koeppel, 2008).

Community assembly operates on both ecological and evolutionary time scales, resulting in contributions from both recent and historical elements. Accordingly, it is difficult to link short-term local processes to global processes that occur over evolutionary time scales and to know at which taxonomic scales these effects become evident. At local geographic scales with no dispersal limitation, environmental heterogeneity, and extinction are expected to be the major drivers of assembly, while across larger scales the effects of dispersal limitation and speciation become more relevant (**Figure 1**, and see a review focused on microorganisms in Whitaker, 2006). A key question that remains undetermined is when (or at which scales) does colonization or *in situ* evolution predominate in the assembly process (Cavender-Bares et al., 2009) because available ecological space is filled either by adaptation of early occupants or by foreign colonization, depending on which occurs first. The observation that many ecologically relevant and biochemically complex traits are phylogenetically conserved (Martiny et al., 2013) seems to support the idea that it is often more feasible for microbial taxa to move than to evolve (Cavender-Bares et al., 2009). That is, some traits that are more similar within clades than among clades might have evolved prior to the current habitat and later arrived by migration of the organisms possessing those traits. For example, a conserved trait like oxygenic photosynthesis has not evolved independently in each habitat; phototrophic microorganisms dispersed and successfully colonized new habitats (Blankenship, 1992). Microbes that have short generation times and are capable of going dormant may have a strong numerical advantage as first colonizers (i.e., priority effects and monopolization; Fukami et al., 2007). Accordingly, serial colonization may yield a pattern of isolation by distance that is not driven by geographic distance *per se*, but driven by historical colonization events (De Meester, 2011). Regretfully, the fossil record, which is the richest source of information on the historical events behind extant communities, is mostly absent for Bacteria and Archaea (but see Schopf and Packer, 1987) and researchers must use extant sequence data for historical reconstructions (e.g., Linz et al., 2007).

## TOWARD A MACROECOLOGY THAT EXTENDS ACROSS THE TREE OF LIFE

### SPECIES ARE NOT INDEPENDENT

Bacterial and archaeal lineages are separated by many millions of years of evolutionary time. For instance, the domain Bacteria is estimated to be approximately 3.5 billion years old (Schopf and Packer, 1987), more than thirty times older than the ancestor of all birds (Padian and Chiappe, 1998). Thus, the amount of evolutionary diversification that has occurred within the bacterial domain will far exceed what is found within groups of plant or animal taxa. This diversification is evident in the astonishing metabolic diversity of bacteria; while nearly all plants have similar requirements for growth, the range of metabolic strategies employed by bacteria is far broader (Kluyver and van Niel, 1956).

Species are not independent entities, but their functional and ecological similarities are shaped by patterns of common ancestry (Felsenstein, 1985). In a hypothetical world in which evolution was rapid, and in which any lineage was unconstrained by dispersal limitations, communities in similar environments would also be similar. However, evolution is often constrained and lineages tend to be restricted in their geographic distribution (Losos, 1996). In order to account for the non-independence of species, a set of phylogenetic tools has recently been developed that aim to bridge the gap between evolutionary and ecological analyses (see a recent review in Cavender-Bares et al., 2009). Thus, ecologists can use such phylogenetic methods to determine: (i) where most of the biological diversity accumulates (Faith, 1992) and how it is intrinsically structured (Webb, 2000), and (ii) how phylogenetic community similarity is distributed along environmental gradients (Lozupone and Knight, 2005). For example, it has been shown for both bacteria and archaea that soil, even with high taxonomic diversity, tends to be less phylogenetically diverse than other habitats such as marine sediments and that salinity is the main driver of phylogenetic community patterns at the global scale (Lozupone and Knight, 2007; Auguet et al., 2010). Thus, incorporating phylogenetic information into macroecology is useful because it allows ecological questions to be addressed in an evolutionary context, the common set of processes that ultimately shapes all biological diversity.

### A MAJOR MACROECOLOGICAL TRANSITION IN STOCHASTIC GEOMETRY?

It is still uncertain whether bacterial and archaeal cells exhibit distinct macroecological patterns from those commonly observed for multicellular eukaryotes which have been the focus of nearly all macroecological research. In general, similar patterns have been documented for bacterial, archaeal, and eukaryotic organisms (Soininen, 2012). Nevertheless, some important differences have been reported for microbial communities: species abundance distributions tend to have more rare taxa (i.e., longer tails, as noted above; Curtis et al., 2006), species-area relationships have lower slopes (*z*-values; Horner-Devine et al., 2004; Lennon and Jones, 2011), and the decrease in community similarity with spatial distance is lower (Hanson et al., 2012; Soininen, 2012). Additionally, a number of classic ecological patterns show conspicuous differences: latitudinal richness gradients do not appear to exist in either marine or soil environments (Ladau et al., 2013), and elevational richness gradients are infrequently observed for microorganisms (Fierer et al., 2011). Often, similar patterns emerge when similar mechanisms operate, while different patterns can be due to distinct mechanisms or to the same mechanisms operating at different spatial, temporal, or taxonomic scales (Levin, 1992). For example, although a general increase in metabolic rate with body mass has consistently been observed across the tree of life, this relationship has been hypothesized to be a function of genome size in prokaryotes and a function of body size in plants and animals; a difference that could contribute to the distinct scaling relationships observed for these groups of organisms (DeLong et al., 2010).

**FIGURE 2:**
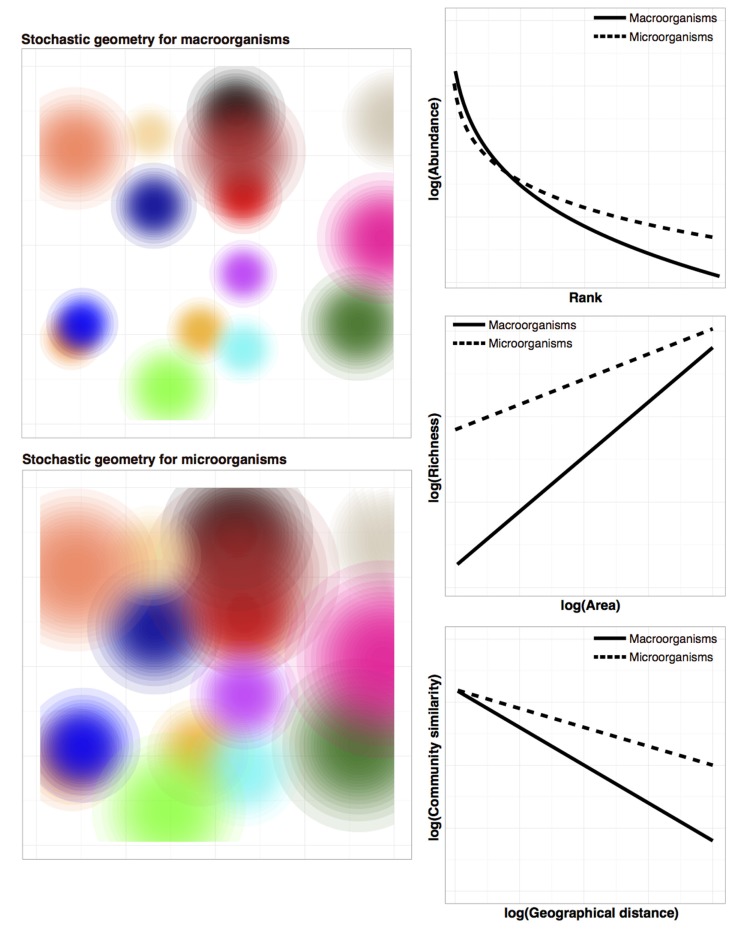
**Model simulation results of the stochastic geometry theory (McGill, 2010) as applied to either macroorganisms (top left) or microorganisms (bottom left)**. The only difference between both simulations is the “dispersal” parameter (i.e., larger spread of the spatial distributions for microbial species). For simplicity, the number of species (represented as different colors) has been set to fifteen for both macroorganisms and microorganisms. Axes represent the two spatial dimensions, while color intensity indicates relative abundance. As explained in McGill (2010), species abundance distributions (top right) are generated by sampling at one point in the spatial grid, species-area relationships (mid right) are created by sampling increasingly large areas, while the decrease of community similarity with spatial distance (bottom right) is derived by sampling areas of the same size at different distances. The tendency of microorganisms to be better dispersers (larger spatial distributions) is sufficient to reproduce the observed qualitative differences of macroecological patterns between macroorganisms and microorganisms. For microbes, the key differences observed for microorganisms versus macroorganisms include: richer species abundance distributions with longer tails of rare taxa (Curtis et al., 2006), species-area relationships with a higher total number of species and with lower slopes (Lennon and Jones, 2011), and a more moderate decay of community similarity with distance (Soininen, 2012). That is, microbial communities would tend to have a higher number of species (richness, or alpha-diversity) but lower turnover (beta-diversity).

Recently, McGill (2010) showed that most predictions about macroecological patterns can be generated by three simple rules regarding the random placement of organisms in space (i.e., stochastic geometry): (i) individuals within a species tend to be spatially clustered, (ii) abundance between species varies (many species are rare and a few are common), and (iii) the spatial distributions of individuals from one species are independent from the distributions of other species (i.e., species interactions are non-existent). Although the first two assumptions appear more reasonable than the third, interspecific spatial independence may indeed be a good statistical approximation in species-rich communities (Wiegand et al., 2012). **Figure 2** shows simulation results from the stochastic geometry model (McGill, 2010) as applied to macroorganisms and microorganisms. All else being equal, the tendency of microbes to have greater dispersal capabilities compared to macroorganisms (represented as larger spatial distributions in **Figure 2** bottom left) is sufficient to reproduce the abovementioned differences reported for the shape of the species abundance distribution, species-area relationship and the decrease of community similarity with distance (see **Figure 2** for details). This simple modeling exercise demonstrates that incorporating the aforementioned microbial idiosyncrasies (in this case, high dispersibility) to existing macroecological models can generate some of the differences in community patterns between micro and macroorganisms observed in the environment.

### A CONCEPTUAL NEUTRAL MODEL FOR MICROORGANISMS

The neutral theory of biodiversity considers communities as open, non-equilibrial assemblages of ecologically equivalent species, with the abundances of individual taxa within communities largely governed by random speciation and extinction events, dispersal and ecological drift (Hubbell, 2001). The publication of Hubbell’s book was controversial among ecologists due to many of the assumptions being considered unrealistic or at least inconsistent with what is known about the natural history of many organisms (Alonso et al., 2006). The most criticized aspect of Hubbell’s theory was the assumption of neutrality. In Hubbell’s model, all individuals of different species in a community are strictly equivalent in their probability of reproduction and death. In his neutral framework, the known and evident differences between species are irrelevant for the prediction of large-scale patterns. Surprisingly, neutral theory predicts observed species abundance distributions, species-area relationships, and community similarity patterns with distance (Hubbell, 2001). Communities may seem neutral because they are complex (i.e., equivalence may occur from non-neutral processes by statistical averaging; Pueyo et al., 2007) with patterns emerging from a statistical process of intricate causalities (Maurer, 1999). Thus, the neutral theory resembles the kinetic theory of gases: it is an ideal theory (i.e., neither ideal gases nor pure neutral communities exist) that does not necessarily encapsulate the messy details of reality (Alonso et al., 2006).

**FIGURE 3:**
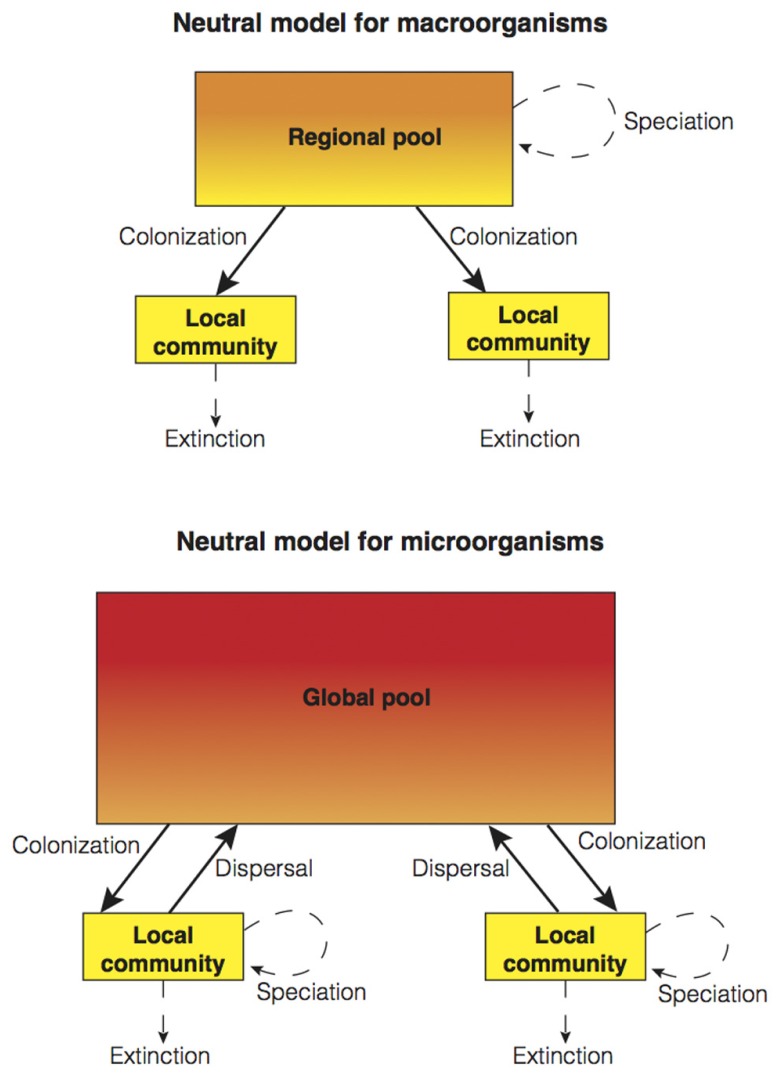
**A schematic representation of two neutral models: Hubbell’s original two-level spatially implicit model (Hubbell, 2001) for macroorganisms (above), and a suggested model for microorganisms (below)**. Both models are based on the same mechanistic processes operating at different scales. The main differences are that, in the neutral model for microorganisms, global scale dynamics become more important with the incorporation of long-distance dispersal to the global pool due to high dispersibility, and high speciation introduced by placing this process at the local scale. As in **Figure 1**, the demarcation of discrete spatial scales is arbitrary.

As originally formulated by Hubbell, neutral theory might seem unsatisfactory to a microbial ecologist (though neutral models have already been applied to microbial communities; Sloan et al., 2006) due to the idiosyncrasies of microbial communities (i.e., high dispersibility and high speciation; see above). Here, we propose that a unified neutral theory composed of two models is required in order to cover the full extent of biological diversity found in both macrobial and microbial communities (see **Figure 3** for a conceptual summary). How do we integrate the seemingly high capacity for bacterial and archaeal dispersal into models of community dynamics? In the modified conceptual model for microbial communities (**Figure 3**), the regional scale is often neglected due to high microbial dispersibility, and the global scale gains preponderance. In Hubbell’s neutral model formulation for macroorganisms (Hubbell, 2001), local and regional scales are connected through unidirectional migration (i.e., colonization from the regional pool to the local community). In contrast, in the microbial model, organisms are allowed to disperse long-distances and thus, be part of the global pool (that is, the rare biosphere or seed bank; Lennon and Jones, 2011) as a result of high dispersibility and dormancy. Hence, individuals may exit local communities through mortality fueling local extinction, or by long-distance dispersal forming part of the global pool. As not all microbial taxa have identical capacities for dispersal and dormancy, regional pools still might exist but at smaller spatial scales. How might we integrate the potentially high rates of microbial speciation into community models? In the conceptual model for microorganisms, speciation takes place at the local scale rather than at the regional scale (**Figure 3**). In addition, the problematic species concept for asexual microorganisms may suggest treating speciation as a continuous process rather than as a discrete process (Rosselló-Mora and Amann, 2001). In Hubbell’s formulation, each individual has a fixed probability to speciate (i.e., point mutation speciation; Hubbell, 2001). To resolve this unrealistic scenario, Rosindell et al. (2010) developed a model of protracted speciation. This speciation process is not instantaneous, as in Hubbell’s original formulation, but gradual (i.e., it takes time for an incipient species to be recognized as new). Protracted speciation has been able to make realistic predictions about the number of rare species, species lifetimes, and speciation rates (Rosindell et al., 2010) and it may be particularly useful when trying to incorporate microbial speciation into community models.

Contrary to neutral theory, niche theory states that every species possesses a unique set of traits that permits adaptation to abiotic and biotic environmental conditions (Hutchinson, 1957). Measuring traits is essential to differentiate between the different ecological and evolutionary processes given that species largely interact within communities based on their traits, and traits tend to reflect the evolutionary history of species. Trait-based approaches will allow us to assess how and why natural communities depart from the predictions based on neutral models that do not consider the mechanisms by which organisms interact with each other and their environment (Shipley et al., 2006).

## CONCLUSION

More than fifty years ago Kluyver and van Niel (1956) demonstrated the contribution of microorganisms to genetics and biochemistry, and discussed the metabolic characteristics that unite all organisms. As the authors responded to the question “What has microbiology offered to general biology?,” one subsequent reasonable question may be: “What can microbial ecology offer to macroecology?” Even though ecologists are incorporating microbes in their research, there is still a significant lag especially in the conceptual and theoretical development.

Understanding the complex and hierarchical structure of biodiversity (the Baroque of Nature as expressed by the ecologist Ramon Margalef; Margalef, 1997) is one of the most challenging tasks of modern science (Solé and Bascompte, 2006). Over the past decade microbial ecologists have generated abundant molecular data from environmental surveys, and now we are able to combine bioinformatics and statistical tools with critical testing of ecological theory in order to integrate microorganisms into the general field of ecology. Ecologists can no longer ignore microbial communities in the development of ecological theory now that we have the tools available to interrogate this long hidden face of diversity.

Here, we encourage microbial ecologists to move beyond Baas Becking’s tenet, everything is everywhere: but the environment selects (Baas Becking, 1934), toward testable theories built upon evolutionary and ecological mechanisms that extend across the tree of life. Incorporating the microbial idiosyncrasies into the conceptual frameworks of macroecology would help to assess the importance of different processes in community assembly and the interplay between ecological and evolutionary time scales. Together, such conceptual approaches would contribute to the unification of microbial ecology and general ecology.

## Conflict of Interest Statement

The authors declare that the research was conducted in the absence of any commercial or financial relationships that could be construed as a potential conflict of interest.
